# Enhanced osseointegration of dental implants with reduced graphene oxide coating

**DOI:** 10.1186/s40824-022-00257-7

**Published:** 2022-03-21

**Authors:** Yong Cheol Shin, Ji-Hyeon Bae, Jong Ho Lee, Iruthayapandi Selestin Raja, Moon Sung Kang, Bongju Kim, Suck Won Hong, Jung-Bo Huh, Dong-Wook Han

**Affiliations:** 1 Department of Cogno-Mechatronics Engineering, College of Nanoscience & Nanotechnology, Pusan National University, Busan, 46241 South Korea; 2 Department of Biomedical Engineering, The University of Texas at Austin, Austin, TX 78712 USA; 3 Department of Prosthodontics, Dental Research Institute, Dental and Life Sciences Institute, Education and Research Team for Life Science on Dentistry, School of Dentistry, Pusan National University, Yangsan, 50612 South Korea; 4 Daan Korea Corporation, Seoul, 06252 South Korea; 5 Bio-IT Fusion Technology Research Institute, Pusan National University, Busan, 46241 South Korea; 6 Dental Life Science Research Institute / Innovation Research & Support Center for Dental Science, Seoul National University Dental Hospital, Seoul, 03080 South Korea

**Keywords:** Titanium, Reduced graphene oxide, Osteogenesis, Bone tissue engineering, Surface coating

## Abstract

**Background:**

The implants of pure titanium (Ti) and its alloys can lead to implant failure because of their poor interaction with bone-associated cells during bone regeneration. Surface modification over implants has achieved successful implants for enhanced osseointegration. Herein, we report a robust strategy to implement bioactive surface modification for implant interface enabled by the combinatorial system of reduced graphene oxide (rGO)-coated sandblasted, large-grit, and acid-etched (SLA) Ti to impart benefits to the implant.

**Methods:**

We prepared SLA Ti (ST) implants with different surface modifications [i.e., rGO and recombinant human bone morphogenetic protein-2 (rhBMP-2)] and investigated their dental tissue regenerating ability in animal models. We performed comparative studies in surface property, in vitro cellular behaviors, and in vivo osseointegration activity among different groups, including ST (control), rhBMP-2-immobilized ST (BI-ST), rhBMP-2-treated ST (BT-ST), and rGO-coated ST (R-ST).

**Results:**

Spectroscopic, diffractometric, and microscopic analyses confirmed that rGO was coated well around the surfaces of Ti discs (for cell study) and implant fixtures (for animal study). Furthermore, in vitro and in vivo studies revealed that the R-ST group showed significantly better effects in cell attachment and proliferation, alkaline phosphatase activity, matrix mineralization, expression of osteogenesis-related genes and protein, and osseointegration than the control (ST), BI-ST, and BT-ST groups.

**Conclusion:**

Hence, we suggest that the rGO-coated Ti can be a promising candidate for the application to dental or even orthopedic implants due to its ability to accelerate the healing rate with the high potential of osseointegration.

**Supplementary Information:**

The online version contains supplementary material available at 10.1186/s40824-022-00257-7.

## Introduction

Shortening the healing period after an implant placement surgery is the most priority in dental implants. In particular, initial osseointegration and subsequent preservation are the most potent factors that promote the healing rate [1, 2]. Osseointegration is defined as an intimate contact between living bone and implant surface without the intervention of fibrous connective tissues, which is a crucial factor in enhancing the long-term clinical success of dental implants [3, 4]. Among other factors, the provided surface of the dental implant plays an important role in the early stage of osseointegration, which has a direct impact on the later development of bone growth in patients [5]. To date, the most widely used materials for dental and orthopedic implants are pure titanium (Ti) and its alloys with excellent mechanical strength, chemical stability, and biocompatibility [6]. Unfortunately, they do not bind directly to bone or actively induce new bone formation because these materials are biologically inert. Indeed, incomplete integration of pure Ti implants with the bone cells and tissues requires a longer interval between surgery and implant loading, ultimately increasing the chance of implant failure [1, 7]. Therefore, many elaborated approaches on surface modification and methods have been developed to improve the effectiveness of osseointegration on the implanted Ti interfaces.

Recently developed techniques have focused on the modification of implant surface properties that critically determine osseointegration during bone healing. For example, surface treatment processes such as Ti plasma-spraying, grit-blasting, acid-etching, anodization, and coating with inorganic calcium phosphate have been suggested to promote healing rates with increased osseointegration, improving the augmentation of surrounding bone [7–9]. Among these surface modification techniques, sandblasted, large-grit, and acid-etched (SLA) treatment has been frequently used as a standard process to modify the surfaces of Ti dental implants due to its successful contribution to clinical performance. However, challenges still remain in improving the surface properties to promote dental tissue regeneration because of the inferior osseointegration of Ti dental implants derived by biological inactivation. To overcome this problem, several strategies have been suggested for the successful assessment of implantation in bone defect sites by employing biomimetic molecular modifications, such as Arg-Gly-Asp (RGD) peptide [10], collagen [11], and growth factors like bone morphogenetic proteins (BMPs) [12]. Although successful integration was clearly established, the steps to coat the implant with biomolecules were not simple, and the utilities in the process required multiple separated bath. Therefore, new technological efforts to modify the implant surface are still being implemented in this field of research.

From a biomaterial point of view, graphene family nanomaterials, including graphene oxide (GO) and reduced GO (rGO), have offered promising technological opportunities, exhibiting inherent superior physical and chemical properties in the coated form of thin films [13]. Notably, these versatile graphitic nanomaterials have been employed in various types of biomedical applications, including bioimaging, biosensing, drug delivery, and tissue engineering, mainly due to the outstanding nature of biocompatibility [14–16]. For example, the potential of rGO has been investigated as a prominent promoter for osteogenic differentiation of human mesenchymal stem cells (hMSCs) [17]; it has been found that the functional group-containing basal planes and edges of rGO actively absorb and interact with surrounding biomolecules to direct cell behaviors. On this, our previous studies have evaluated clear cellular responses specifically on the enhanced osteogenesis by utilizing graphene-based nanomaterials [18], and the next step is to evaluate an osteogenic efficacy in practical application using in vivo animal models. It has been reported that recombinant human BMP-2 (rhBMP-2), a member of the transforming growth factor β superfamily, is involved in the differentiation of MSCs into osteoblasts, biosynthesis of bone matrix, and stimulation of the ossification process by regulating bone induction cascade factors [19]. Unfortunately, those growth factors have been also reported to show their presumable adverse effects, such as ectopic bone formation and postoperative inflammation and tumorigenesis [20]. Therefore, the development of alternative strategies in dental implant applications may be an unmet urgent need.

In this study, as a novel technique for promoting dental tissue regeneration, we developed a simple and robust process to endow conventional SLA Ti (ST) implants with bioactive surface by coating in vitro confirmed rGO. In a systematic experimental condition, the concentration of rGO in the coating process for rGO-coated ST (R-ST) implants was optimized via the series of physicochemical characterizations and in vitro cytocompatibility tests of the R-ST discs, prior to the comparative in vivo studies. Unique regular and ultrafine-grained surface of the R-ST disc was carefully explored by microscopic grain size analysis and the homogenous surface properties of ultrathin rGO-coated substrates were evaluated on the ST discs. Finally, in vivo osseointegration efficiency of four different experimental groups, including ST (i.e. SLA Ti, control), BI-ST (i.e. rhBMP-2-immobilized ST), BT-ST (i.e. rhBMP-2-treated ST), and R-ST, was examined by implanting them in a beagle dog mandible.

## Materials and methods

### Materials

The ST discs and implant fixtures (CP Ti, Grade II, ASTM F67) were kindly provided from Cowellmedi Co. Ltd. (Busan, Korea) for in vitro and in vivo studies, respectively. Their surfaces were modified with rGO or rhBMP-2 (Cowellmedi Co. Ltd.). GO was purchased from Graphene Laboratories Inc. (Ronkonkoma, NY). (3-aminopropyl)triethoxysilane (APTES, 99%) and ethanol were purchased from Sigma-Aldrich Co. (St Louis, MO). Epigallocatechin-3-*O*-gallate (EGCG, ≥96%) was kindly supplied by BMG Inc. (Kyoto, Japan) and de-ionized (DI) water were purchased from Samchun Chemical Co. (Seoul, Korea). All the solvents used were of analytical grade and all the chemicals received were used without further purification.

### Preparation of surface-modified ST discs and implants

**Scheme 1:**
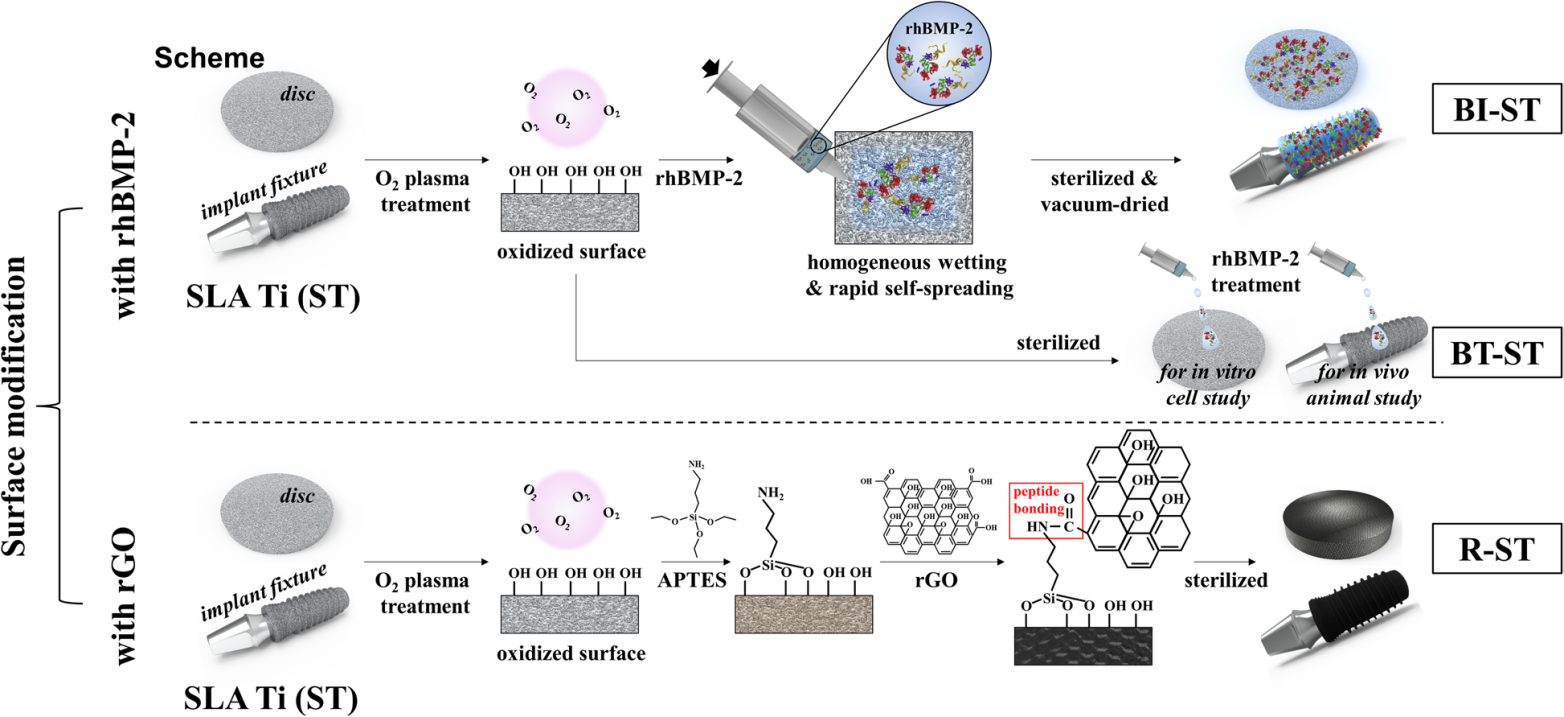
Surface-modification strategies for SLA Ti (ST) discs and implant fixtures with rhBMP-2 immobilization (BI-ST), rhBMP-2 treatment (BT-ST), and rGO coating (R-ST)

The surface of commercially available ST discs and implant fixtures was modified by different functionalization process as shown in Scheme 1. For preparing the R-ST discs and fixtures, their surface was initially oxidized and cleaned with O_2_ plasma treatment at room temperature (RT) for 1.5 h and then immersed in 3% solution of APTES in ethanol for 1.5 h to introduce positive amine groups on the surface. After washing with DI water thrice, rGO solution was deposited on each surface via peptide bonds between the carboxyl groups of rGO and terminal amino groups of APTES. rGO had been converted from GO by heating in EGCG solution as described elsewhere [21]. Differently concentrated R-ST discs (10, 100, and 1000 μg/mL) were prepared in order to optimize the coating concentration of rGO on the surface (see Table S1). In the case of the BI-ST discs and fixtures, they were prepared according to the similar method as described in the previous study [22]. In brief, 15 μL aliquot of an rhBMP-2 solution (1 mg/mL) was pipetted onto the center of the sample surfaces. Homogeneous wetting of the surfaces was achieved by the rapid self-spreading of the rhBMP-2 solution across the dry ST surfaces by capillary forces. Finally, the samples were frozen and dried under sterile conditions (at − 40 °C), and then vacuum dried at maximum 20 °C. To confirm rhBMP-2 immobilization, the BI-ST discs were incubated with primary rabbit polyclonal BMP-2 antibody (1:250; Abcam, Cambridge, MA) in Dulbecco’s phosphate-buffered saline (DPBS, pH 7.4; Gibco BRL, Rockville, MD) containing 2% bovine serum albumin (BSA; Sigma-Aldrich Co.) at RT for 2 h. After rinsing thoroughly, secondary tetramethylrhodamine isothiocyanate (TRITC)-labeled rabbit anti-human IgG (1:500, 2% BSA solution in DPBS; Abcam) was added and reacted at RT in the dark for 1 h. Imaging was carried out using an upright fluorescence (FL) microscope (BX51, Olympus, Osaka, Japan) with a digital camera (Olympus) and the red FL signals from the micrographs were quantified using ImageJ software (National Institutes of Health, Bethesda, MD). For comparison in in vitro and in vivo studies, the BT-ST discs (150 ng/mL of rhBMP-2 treatment for in vitro study) and implants (15 μg/mL of rhBMP-2 treatment upon implant placement) were also prepared respectively.

### Physicochemical characterizations

The surface topography of the R-ST was measured by atomic force microscopy (AFM; NX10, Park Systems Co., Suwon, Korea), followed by image analysis using XEI Software (Park Systems Co.). Water contact angle analysis was carried out by a contact angle goniometer (EasyDrop, model FM40Mk2, Krüss, Hamburg, Germany) using a drop-shape analysis program. Raman and X-ray photoelectron spectra (XPS) of samples were collected using Raman spectrometer (Micro Raman PL Mapping System, Dongwoo Optron Co., Ltd., Gwangju, Korea) and x-ray photoelectron spectrometer (AXIS Supra, Kratos Analytical, UK), respectively. The base pressure in the XPS chamber was 5 × 10^− 10^ mbar. A wide scan was performed correcting the binding energies with C 1 s as reference energy (C 1 s = 284.8 eV). Fourier transform infrared (FTIR) spectra were collected by an FTIR spectrophotometer (Nicolet 560, Nicolet Co., Madison, WI). All spectra were recorded in absorption mode in the wavelength range of 1000-4000 cm^− 1^ with a resolution of 4.0 cm^− 1^ and 16-times scanning. Electron backscattered diffraction (EBSD) analysis was carried out by using the TEAM™ EBSD Analysis System combined with energy-dispersive X-ray (EDAX) to evaluate the texture and grain size of the samples setting the voltage at 20 kV and current at 6.0 nA.

### Protein adsorption, cell attachment and cell proliferation assays

hMSCs were purchased from Lonza (Walkersville, MD) and used between passages 5 and 7. Cells were routinely cultured in MSC basal media (BM, Lonza), supplemented with 10% MSC growth supplement, 2% L-glutamine, and 0.1% GA-1000, containing a 1% antibiotic antimycotic solution (including 10,000 units penicillin, 10 mg streptomycin, and 25 μg amphotericin B per mL, Sigma-Aldrich Co.) in a humid incubator maintaining an atmosphere of 5% CO_2_ and 37 °C.

To quantify the surface protein adsorption on the samples, the protein concentration after incubation of the samples with DPBS or media was determined by the bicinchoninic acid (BCA) assay. Briefly, disc samples were prepared and placed in a 24-well plate. The samples were washed three times with DI water and dried overnight under vacuum at RT. Then, samples were incubated with DPBS containing 10% fetal bovine serum (FBS, Welgene, Daegu, Korea), MSC BM (without any supplements or FBS), and complete media (BM with supplements and 10% FBS) for 24 h at 37 °C. Samples were washed with DI water thrice, and the protein concentration was measured by using Micro BCA™ Protein Assay kit (Pierce Biotechnology, Rockford, IL) following the manufacturer’s protocol. To exclude the effect of BMPs immobilized on the surface of the BI-ST discs, the discs were pretreated with 1% Triton X-100 solution (Sigma-Aldrich Co.) and then thoroughly rinsed with DPBS to remove residual surfactant. For immunofluorescence staining of adsorbed proteins on each disc after incubation in DPBS with 10% FBS, all discs were treated with fluorescein isothiocyanate (FITC)-labeled goat anti-rabbit IgG (at 1:500, 2% BSA in DPBS) at RT in the dark for 1 h as described in our previous study [23]. Immunofluorescence imaging was implemented using an upright FL microscope with a digital camera as described above.

To assess cell attachment, hMSCs (1 × 10^5^ cells/mL) were seeded onto different Ti discs and cultured for 6 h. The non-adherent cells were removed by washing with DPBS three times. The cell number was quantified as a percentage of cell attachment using optical microscope images captured at different magnifications (inverted Leica DMIL microscope, Leica Microsystems, Wetzlar, Germany). A cell counting kit-8 (CCK-8) assay (Dojindo Laboratories, Kumamoto, Japan) was carried out to evaluate the proliferation of hMSCs. In brief, the cells were seeded onto the ST, BI-ST, BT-ST, and R-ST discs at a concentration of 1 × 10^4^ cells/mL, and cultured in the BM. On 1, 7, 14, and 21 days, the cells were washed with DPBS twice and incubated with CCK-8 solution for 2 h in the dark at 37 °C. The cell proliferation on different Ti discs was assessed by measuring the absorbance at 450 nm using an ELISA reader (SpectraMax® 340, Molecular Devices, Sunnyvale, CA).

### Alkaline phosphatase (ALP) activity assay and alizarin red S (ARS) staining

ALP activity, an early marker of osteoblast differentiation, was characterized using a commercial ALP assay kit (Abcam) according to the manufacturer’s protocol. hMSCs were seeded onto the ST, BI-ST, BT-ST, and R-ST discs at a density of 1 × 10^4^ cells/mL, and incubated at 37 °C. The cells were washed twice with DPBS and lysed using an ALP assay buffer with 1 h incubation at 37 °C. An 80 mL of lysate was mixed to a 50 μL of freshly prepared *ρ*-nitrophenyl phosphate solution (1 mM) and incubated at 37 °C for 1 h. A 20 μL of stop solution was added to quench the reaction. The ALP activity was calculated as the amount of *ρ*-nitrophenol (nM) divided by the volume of sample (mL) and the reaction time (min).

The extracellular mineralization was monitored by ARS staining. hMSCs were seeded onto the ST, BI-ST, BT-ST, and R-ST discs at a density of 1 × 10^4^ cells/mL, and cultured for 1 to 21 days. The cells were washed twice with DPBS, fixed with 2% paraformaldehyde (Sigma-Aldrich Co.), and stained using a 40 mM ARS solution (pH 4.2, Sigma-Aldrich Co.) for 20 min. To quantify mineralized nodule (%), the stained ARS within cells was extracted by adding a 10% acetic acid solution for 30 min with constant agitation and then neutralized with 10% ammonium hydroxide solution. The absorbance was recorded at 405 nm using an ELISA reader.

### RNA isolation and real-time quantitative reverse transcription polymerase chain reaction (qRT-PCR)

For real-time qRT-PCR analysis, hMSCs were seeded onto the ST, BI-ST, BT-ST, and R-ST discs at a density of 5 × 10^4^ cells/mL, and cultured for 14 days. Then, the cells were dissociated with 0.05% trypsin-EDTA (Invitrogen, Carlsbad, CA) by mild pipetting. Total RNA was extracted from the detached cells using TRIzol reagent (Invitrogen) and an RNeasy Mini Kit (Qiagen, Grand Island, NY). SuperScript III First-Strand cDNA Synthesis System (Invitrogen) was further used to synthesize first-strand cDNA from total RNA according to the manufacturer’s instructions. The mRNA expression of specific genes was then determined by real-time qRT-PCR using the total first-strand cDNA as the template and Power SYBR Green PCR Master Mix (Applied Biosystems, Carlsbad, CA). The sequences of the primers for runt-related transcription factor 2 (RUNX2), osteocalcin (OCN), osteopontin (OPN), Vinculin, and β-actin are shown in Table S2. The expression level of β-actin was used as an endogenous normalizer and the relative expression levels were calculated using the ^-ΔΔCt^ method.

### Immunocytochemistry

For immunofluorescence staining, hMSCs were seeded onto the ST, BI-ST, BT-ST, and R-ST discs at a density of 5 × 10^4^ cells/mL, and cultured for 14 days. After incubation, the cells were fixed with 4% formaldehyde (Sigma-Aldrich Co.) for 15 min at RT, permeabilized with 0.2% Triton-X 100 for 5 min, and then blocked with a 2% bovine serum albumin (GenDEPOT, Barker, TX) solution in DPBS for 30 min. To immunostain OCN, the cells were incubated with primary mouse monoclonal antibody to OCN (1:250 dilutions, Abcam) overnight at 4 °C. Subsequently, donkey anti-mouse IgG NorthernLights NL493-conjugated secondary antibody (1:200 dilutions, Abcam) was treated and then reacted at RT in the dark for 2 h. The nuclei were counterstained with 4′,6-diamidino-2-phenylindole (DAPI, 0.3 μM in DPBS, Sigma-Aldrich Co.) at RT for 30 min. The immunofluorescence images were obtained under an upright FL microscope with a digital camera. The green FL signals from the micrographs were quantified using ImageJ software (National Institutes of Health) to compare the relative FL intensity of OCN-positive areas.

### Surgical procedures for animal study and removal torque test

A total of 40 implants with 8.0 mm in length and 3.3 mm in diameter were used for in vivo animal study. Six male beagle dogs (mean age 24 months, mean weight 15 kg) were randomly chosen and six or eight implants in total were installed per animal. All the animals were provided with a soft food diet and free water access. All animal experiments related to surgical procedures and treatments were approved by the Animal Experiment Ethics Committee of Seoul National University and performed in accordance with the Animal Care and Use Committee guidelines (SNU-160923-1).

Initially, surgery was performed to remove mandibular teeth from the experimental animals. The animals were anesthetized by intramuscular injection of atropine sulfate (0.05 mg/kg; Dai Han Pharm Co., Seoul, Korea), followed by isoflurane (Choongwae Co., Seoul, Korea). A 1 mL of 2% lidocaine HCL and 1:100,000 epinephrine (Yu-Han Co., Gunpo, Korea) solution was injected into the extraction area. The premolar and first molar teeth were extracted bilaterally. Subsequently, the extraction area was closed using a sterile synthetic absorbable suture material, 4-0 Vicryl® (Johnson & Johnson, New Brunswick, NJ). When the mandibular extraction area was completely healed after an 8 weeks-period of recovery, a second surgery was carried out to place the implants into the alveolar ridge. The animals were anesthetized following the procedure as described for the teeth extraction. The alveolar ridge was trimmed into a flat ridge and drilled into a hole for implant placement. Each experimental implant was applied randomly (6 implants per animal, *n* = 6 per group) in the edentulous mandibular alveolar ridge and subsequently, the mucoperiosteal flaps were sutured. Intravenous injection of cefazolin (20 mg/kg) was carried out at 48 h of postoperation. Plaque control was managed with daily flushing of the oral cavity with 2% chlorhexidine gluconate. The animals were fed with a soft diet for 2 weeks, followed by a conventional diet in the remaining periods. Intravenous injection of concentrated sodium pentobarbital (Euthasol, Delmarva Laboratories Inc., Midlothian, VA) was performed to sacrifice the animals at 8 weeks of postsurgery. Following euthanasia, a total of 30 block sections of implants, alveolar bone, and surrounding mucosa were harvested from the experimental animals and fixed in a 10% neutral buffered formalin solution (Sigma Aldrich Co.) for the analyses.

With the same procedure as described for the teeth extraction and placement, each implant specimen was installed randomly (8 implants per animal, *n* = 4 per group) in the mandibular alveolar ridge. Two animals were sacrificed at postoperative 8 weeks and then the alveolar ridge placed with implants was immediately processed for the measurement of the maximum removal torque (MRT) with a torque testing machine (CME, Técnica Industrial Oswaldo Filizola, SP, Brazil). The maximal force was measured by removing the specimens via an anti-clockwise rotation and the mean MRT values were calculated for each group.

### Micro-computed tomography (μ-CT) analysis

New bone formation in the peri-implant area was evaluated using μ-CT analysis after 8 weeks of implantation. The specimens were sealed with Parafilm M® (Bemis Company, Inc., Neenah, WI) to prevent drying during the scanning procedure. All the specimens were scanned using a bromine filter (0.25 mm; Skyscan-1173, version 1.6, Bruker-CT, Kontich, Belgium) with the parameters of 60 μA intensity, 130 kV energy, and 7.10 μm-pixel resolution. The images were processed by the NRecon reconstruction software program (Bruker-CT). The width of the region of interest (ROI) in the treated mandibular surgical area was 1.0 mm around the implant and the height was 4.0 mm vertically above the implant platform. New bone volume (NBV; mm^3^) was estimated from the volume occupied by new bone within ROI.

### Histomorphometric analysis

After analyzing μ-CT, the specimens were cleaned thoroughly and dehydrated using ethanol with a gradual increase in concentration from 70 to 100%. The dehydrated specimens were subsequently embedded in acrylic resin (Technovit 7200, Heraeus Kulzer, Hanau, Germany) and then sectioned longitudinally at the center of each implant using a diamond cutting system (EXAKT 300 CP, Norderstedt, Germany). The thickness of the final slides was grounded from the initial 400 μm to the final 40 ± 5 μm by a grinding system (EXAKT 400CS). Goldner Trichrome staining was accomplished to examine the newly regenerated bone tissue. The images of the slides were captured at 40 × magnification using an optical microscope (Olympus) connected with a charge-coupled device digital camera (SPOT Insight 2Mp, Diagnostic Instruments, Sterling Heights, MI) with an adapter (U-CMA3, Olympus). The captured images were further processed using an image-analyzing software (IMT i-Solution Inc., Coquitlam, BC, Canada). Bone-to-implant contact (BIC) ratio (%) was measured from the length of bone contact divided by the total length of the three upper threads. Intra-thread bone density (ITBD; %) was estimated from the areas occupied by the new bone divided by the total area between three upper threads of the implant [24].

### Statistical analysis

All experimental results are presented as the mean ± standard deviation (SD). The statistical analyses were performed using a software R (version 3.1.3). The data were analyzed using a one-way analysis of variance (ANOVA; SAS Institute Inc., Cary, NC), followed by a Bonferroni test for multiple comparisons. Statistical analysis for in vivo results was performed using the Kruskal–Wallis one-way ANOVA and the Mann–Whitney U test. Values of *p* < 0.05^a^, *p* < 0.01^b^, and *p* < 0.001^c^ were considered statistically significant (*n* = 6 for physicochemical measurements and in vitro cell study; *n* = 4 or 6 for in vivo animal study).

## Results

### Optimization of rGO coating and confirmation of rhBMP-2 immobilization on Ti surface

To optimize the coating condition of rGO, the freshly cleaned Ti discs were coated with a range of rGO concentrations (10, 100, and 1000 μg/mL) and each surface property was characterized (Table S1 and Fig. S1). The increase in the applied rGO concentration resulted in increased surface roughness (arithmetic average roughness, R_*a*_) and decreased contact angles of water. hMSCs were separately cultured on the ST and R-ST discs to assess cellular behaviors, such as cell attachment, cell proliferation, ALP activity, and extracellular mineralization. Interestingly, the cellular behaviors on the R-ST groups were found to be substantially promoted regardless of the rGO concentrations as compared to the control groups (ST). Meanwhile, the R-ST discs coated with an rGO solution of 100 μg/mL exhibited the most outstanding surface for favorable cell adhesion, growth, and osteogenic differentiation. Thus, the R-ST samples coated with 100 μg/mL of rGO were employed in the following studies.

For the immobilization of rhBMP-2 on the ST disc surface, the surface was homogeneously wetted by the rapid self-spreading of rhBMP-2 solution by capillary forces, followed by freezing at − 40 °C and vacuum drying. Immunofluorescence intensity and imaging revealed that rhBMP-2 was spatially uniform and evenly distributed throughout the entire ST surface (Fig. S2A and B).

### Physicochemical characterizations of ST and R-ST

**Fig. 1:**
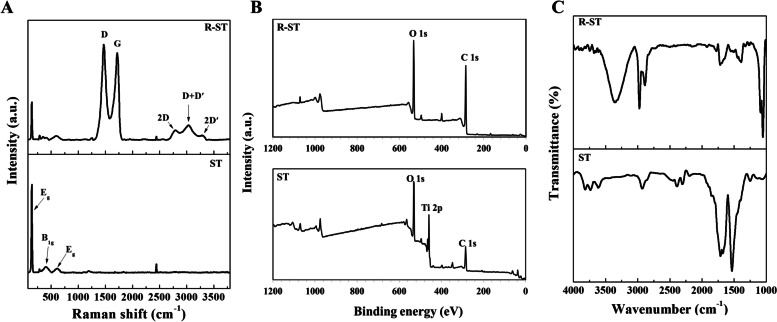
Spectral analysis of ST and R-ST surfaces. **A** Raman, **B** XPS and **C** FTIR spectra of ST and R-ST discs

**Fig. 2:**
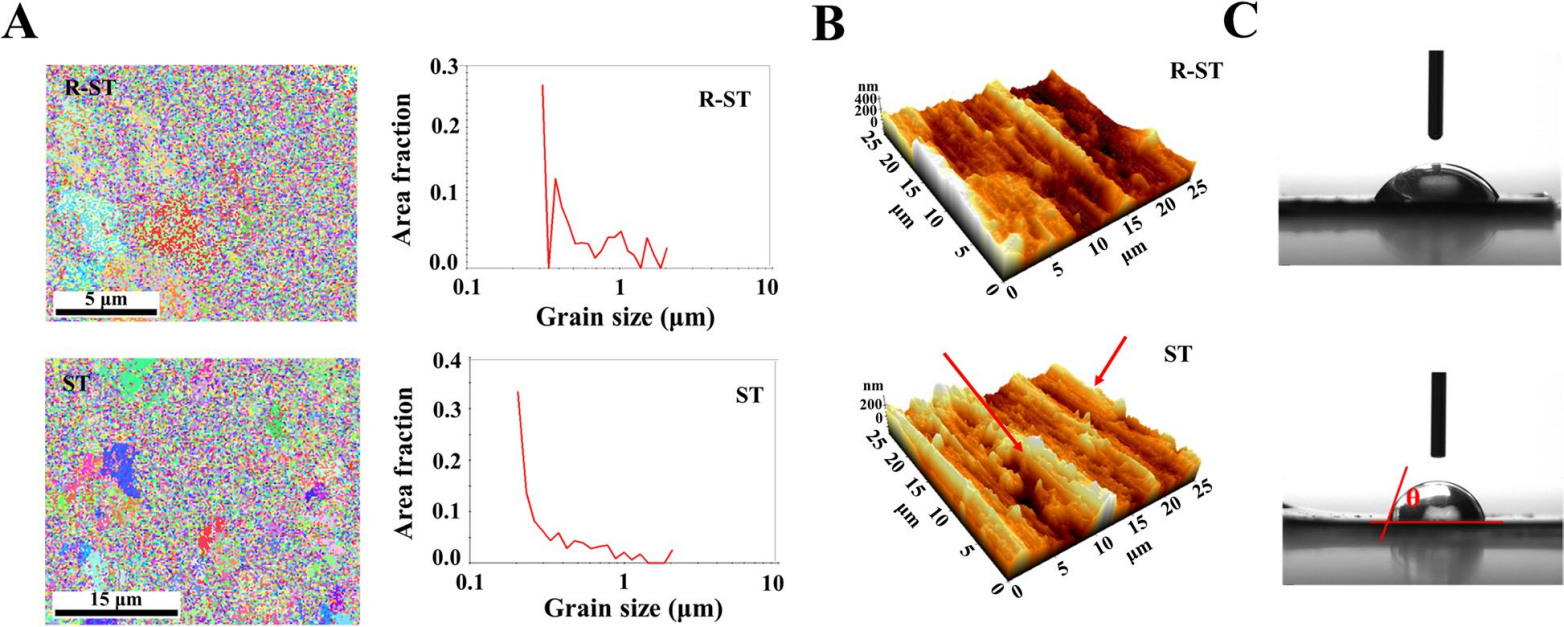
**A** EBSD mapping images, **B** AFM images, and **C** water contact angles (θ) of ST and R-ST discs

We further characterized physicochemically the R-ST samples to investigate its surface properties through spectral, diffraction, and microscopic analyses (Figs. 1 and 2). The Raman spectrum of intact ST disc displayed specific peaks near 144 and 620 cm^− 1^, which can be due to O–Ti–O symmetric stretching and symmetric bending vibrations, respectively (Figs. 1A and S3A). On the other hand, the Raman spectrum of the R-ST represented the characteristic peaks for Ti and rGO with the typical stretching vibration of D (1467 cm^− 1^) and G (1730 cm^− 1^) bands. In addition, the 2D, D + D′, and 2D′ bands near 2780 cm^− 1^, 3166 cm^− 1^, and 3393 cm^− 1^ were also observed [25]. The estimated intensity ratios (I_D_/I_G_ and I_2D_/I_G_) of the D, 2D, and G bands for the R-ST were 1.82 ± 0.09 and 0.37 ± 0.04, respectively, which was well consistent with the previous studies for rGO [26]. As shown in Fig. 1B, XPS spectroscopy was performed to examine the surface chemistry of the R-ST. The ST disc exhibited a characteristic Ti 2p peak at 460 eV, which corresponds to Ti^4+^ [27]. Also, the spectrum had O 1 s and C 1 s peaks at 530 eV and 285 eV, respectively. The R-ST disc reflected O 1 s and C 1 s peaks by the structural formation of the coated rGO on the Ti surface. On the other hand, the spectrum appeared with no trace of the Ti 2p peak. The overall survey of the XPS spectra well agreed with the other previous report [28]. The robust surface coating of rGO on the ST surface was also confirmed by the FTIR spectroscopy (Figs. 1C and S3B). A noticeable band of carboxyl O–H from rGO was observed in the ranges of 3000-3500 cm^− 1^ [29], and the characteristic bands were observed near 2900 cm^− 1^, assigned to the asymmetric and symmetric C–H stretching from the rGO [30]. On the other hand, the absorption band related to Ti–OH bending vibration was shown at around 1600 cm^− 1^ [31]. These results substantiate that the rGO coating on ST surface was successfully performed in a reliable manner.

Moreover, according to EBSD map analysis (Fig. 2A), the average grain size of the ST and R-ST discs was 0.38 ± 0.17 μm and 0.24 ± 0.10 μm, respectively, indicating that the R-ST discs possess a regular and ultrafine-grained surface. The visualized AFM images of the ST and R-ST discs indicate representative surface topographies in the selected areas (Fig. 2B). The quantitative R_*a*_ was extracted as 79.3 ± 0.9 nm and 87.1 ± 1.8 nm for the ST and R-ST discs, respectively, and the surface contact angles of the ST and R-ST were 70.5 ± 1.9° and 53.6 ± 11.9°, respectively (Fig. 2C and Table S1).

### Protein adsorption and in vitro cellular behaviors of hMSCs on modified Ti discs

**Fig. 3:**
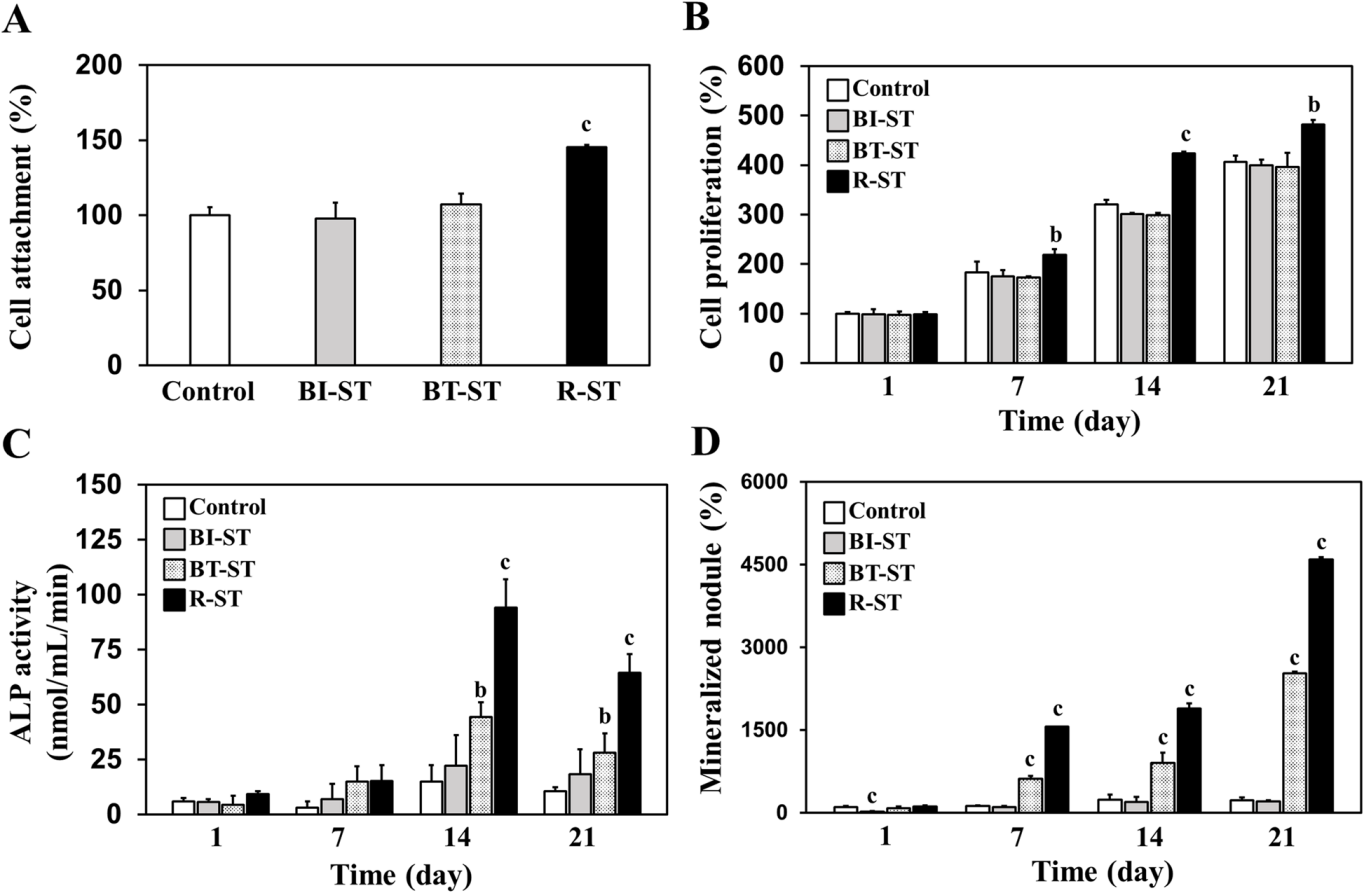
**A** Attachment, **B** proliferation, **C** ALP activity, and **D** mineralization of hMSCs cultured on control (ST), BI-ST, BT-ST, and R-ST discs (*p* < 0.01^b^ and *p* < 0.001^c^, *n* = 6)

The ability to absorb exogenous proteins onto the substrate surface plays a pivotal role in cell attachment and corresponding cell growth. Hence, prior to assessing cellular behaviors on the Ti discs, the adsorption of proteins on each sample surface was analyzed quantitatively and observed by immunofluorescence staining (Fig. S4). Notably, the concentration of absorbed proteins on the R-ST discs was significantly increased as compared to the ST (control) and BI-ST discs regardless of the media composition. It is known that the surface oxygen-containing functional groups triggered the increased adsorption rate of the serum proteins within culture media [32, 33]. This advantageous surface property of the R-ST disc allows the strengthened surface in absorbing the exogenous proteins to elicit efficient interactions with the cultured cells. In the following, to verify the cell-surface interactions, we investigated in vitro cellular behaviors of hMSCs on the ST, BI-ST, BT-ST, and R-ST discs, including cell attachment, proliferation, ALP activity, and extracellular mineralization. It should be noted that the hMSCs cultured on the R-ST discs obviously represented improved cellular interactions compared to those on other groups (Fig. 3). These results could be partially explained by the fact that the rGO-coated surfaces can effectively enhance cell proliferation as well as initial cell attachment [33, 34]. Moreover, R-ST surfaces can provide favorable microenvironments to direct the desired osteogenic responses of hMSCs, as demonstrated in our previous studies [2, 17, 33].

### Expression of osteogenesis genes and proteins in hMSCs on the modified Ti discs

**Fig. 4:**
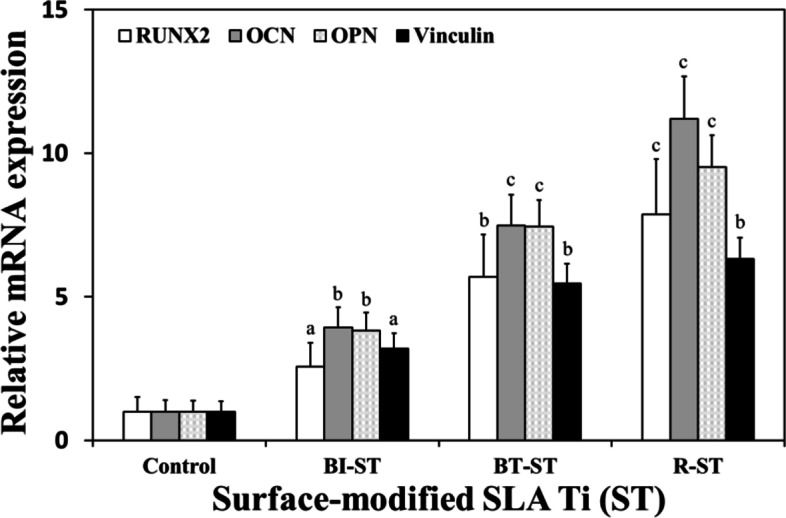
mRNA expression levels of osteogenic markers such as RUNX2, OCN, OPN, and Vinculin in hMSCs on control (ST), BI-ST, BT-ST, and R-ST discs (*p* < 0.05^a^, *p* < 0.01^b^, and *p* < 0.001^c^, *n* = 6)

To explore the underlying mechanism for the spontaneously promoted osteogenic differentiation of hMSCs on the ST, BI-ST, BT-ST, and R-ST discs, the expression of osteogenesis-related genes, such as RUNX2, OCN, OPN, and Vinculin was examined using real-time qRT-PCR. As shown in Fig. 4, regarding the mRNA expression levels of the four established osteogenic differentiation markers, the surface-modified discs significantly upregulated the mRNA expression levels of all the osteogenic markers compared to that on the ST (control). OCN, known as bone γ-carboxyglutamic acid-containing protein, was identified at an early stage in the bone formation process to ensure a calcium-binding, which is secreted solely by osteoblasts [35]. Notably, the cells cultured on the surface of the R-ST discs showed significantly increased mRNA expression of OCN, which was around 1.5-fold and 11.2-fold higher than those cultured on the BT-ST and ST discs, respectively. OPN, bone sialoprotein I, is recognized as one of the factors that strongly bind to various types of calcium-based biominerals in bones and teeth [36]. Significant upregulation of OPN expression was found up to 9.5-fold on the R-ST discs directly, compared to the control (ST). For the further validation of osteogenesis, the mRNA expressions of RUNX2 and Vinculin were quantified. Significantly increased expressions were clearly observed in the cells cultured on the surface-modified ST at 14 days. As well known, RUNX2 is a key transcription factor involved in early osteodifferentiation as the master gene of bone formation, but is not essential for late osteoblast differentiation [37]. On the contrary, Vinculin is a ubiquitously expressed actin-binding protein frequently used as a marker for both focal adhesion and adherens junctions [38]. Therefore, as the increased expression of those genes might simultaneously regulate mineralization and transcription in hMSCs on rGO-coated bioactive surfaces, we postulate that the spontaneous osteogenic differentiation can be attributed to the proteins expressed by these genes.

**Fig. 5:**
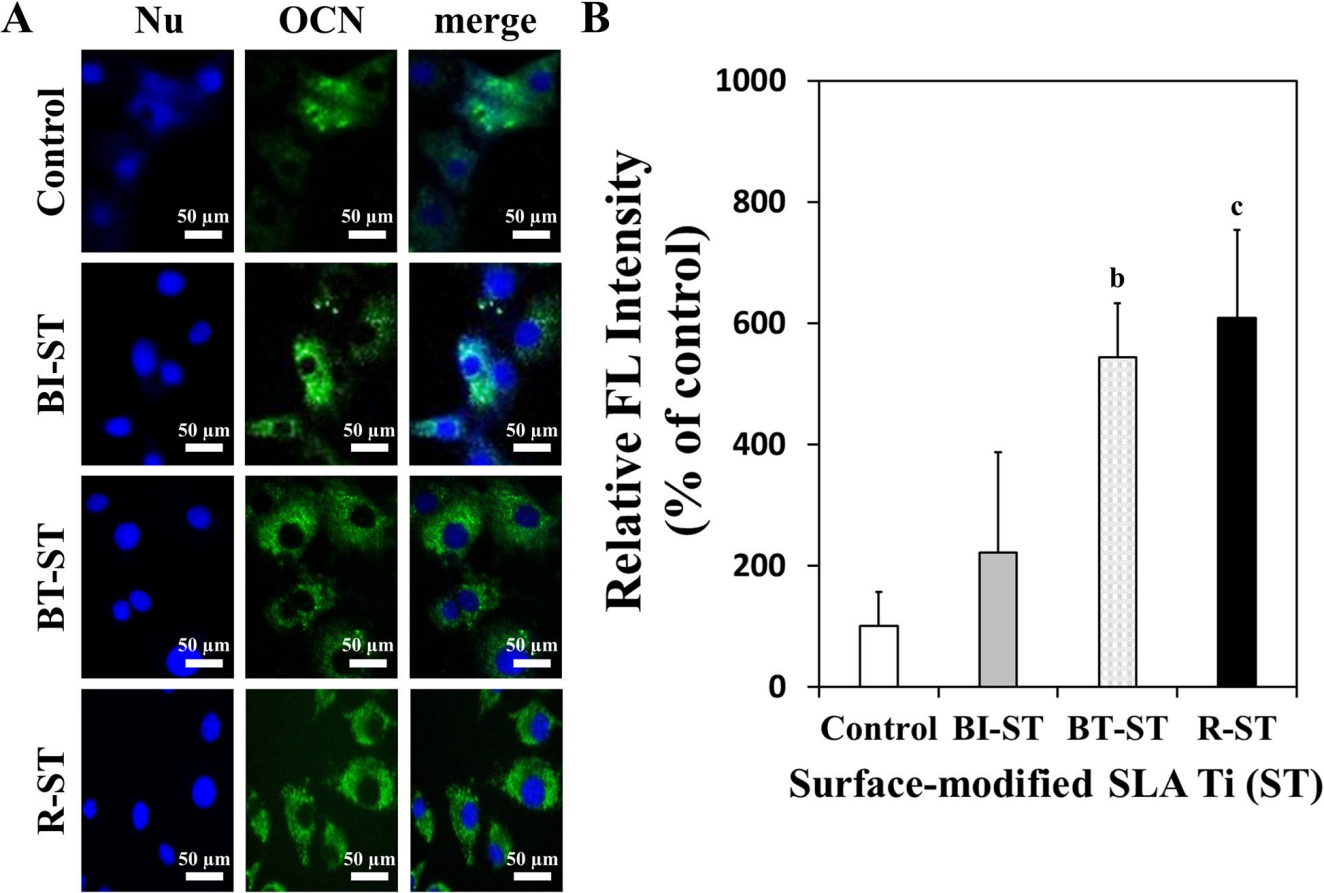
Immunofluorescence staining of OCN (**A**) and relative FL intensity (**B**) in hMSCs on control (ST), BI-ST, BT-ST, and R-ST discs (Nu: nucleus; blue FL from DAPI and green FL from NorthernLights NL493; *p* < 0.01^b^ and *p* < 0.001^c^, *n* = 6)

In addition to the earlier evaluation, immunofluorescence analysis was performed to investigate the expression of OCN protein, which is associated with matrix maturation and calcification as one of the representative markers for osteogenesis [35]. Immunofluorescence staining for OCN demonstrates that the R-ST and BT-ST discs appreciably upregulated the expression of OCN in hMSCs at 14 days of incubation in BM without any osteogenic factors (Fig. 5). In contrast, the control (ST) and BI-ST disc showed a relatively lower expression of OCN. The merged images of nucleus and OCN indicate obvious differences between the ST and R-ST discs (Fig. 5A). This observation was confirmed by the relative FL intensity to show that the green FL signal from the micrograph of the R-ST or BT-ST disc was significantly higher than that of the control (Fig. 5B). This collective set of results fully agreed with that of qPT-PCR (Fig. 4) and implied that the R-ST possessed the remarkable potential to promote the spontaneous osteogenic differentiation of hMSCs. We believe that the rGO coatings would be potential candidates for promising scaffolds in bone tissue engineering, stimulators for osteogenic differentiation of SCs, and components of implantable devices, due to their excellent biocompatibility and bioactivity.

### In vivo performance evaluation of R-ST using removal torque and μ-CT analyses

After the flattening and trimming of alveolar bone and forming drilled holes into the alveolar ridge, the in vivo performance was accessed by transplanting experimental implants on the defective sites (Fig. S5A-C). We observed that all the experimental animal groups survived without any complications during the surgical procedures. Volumetric analyses were performed to evaluate NBV using μ-CT images 8 weeks after implantation with the ST, BI-ST, BT-ST, and R-ST implant fixtures (Fig. S5D and E). The estimated NBV (mm^3^), BIC (%), and ITBD (%) values for each group were summarized in Table 1. The R-ST group exhibited predominantly the highest value of NBV compared to the other groups. The ROI around the implant site was further analyzed for the subsequent histological and histomorphometric evaluation. Moreover, the results from the removal torque analysis of implant fixtures with different surface modifications in the canine alveolar ridge are described in Table 2. At 8 weeks of postsurgery, the highest mean value of MRT was found in the R-ST group, while the difference between the control (ST) and BI-ST group was not statistically significant. Removal torque has been used as a biomechanical measure of anchorage, or endosseous integration; greater torque required to remove implants may be interpreted as an increase in the strength of bony integration [39].

**Table 1:** The volumetric and histomorphometric parameters estimated from the μ-CT images of the upper three threads around the ROI (*p* < 0.05^a^, *p* < 0.01^b^, and *p* < 0.001^c^, *n* = 6)

Implant Group	Variable		
	NBV^*^ (mm^3^)	BIC^*^ (%)	ITBD^*^ (%)
Control (ST)^**^			
Mean	22.26	63.26	60.96
SD	4.05	4.70	4.74
BI-ST^**^			
Mean	27.58	70.20 ^a^	65.33
SD	4.16	5.07	5.11
BT-ST^**^			
Mean	32.42^a^	79.34^b^	77.37^b^
SD	3.07	6.09	5.18
R-ST^**^			
Mean	41.84^b^	89.45^c^	88.58^c^
SD	4.27	5.32	5.83

*NBV New Bone Volume, *BIC* Bone-to-Implant Contact, *ITBD* Intra-Thread Bone Density

**ST: SLA Ti; BI-ST: rhBMP-2-immobilized ST; BT-ST: rhBMP-2-treated ST; R-ST: rGO-coated ST

**Table 2:** The removal torque value (*p* < 0.05^a^ and *p* < 0.01^b^, *n* = 4)

	Control (ST)^*^	BI-ST^*^	BT-ST^*^	R-ST^*^
MRT^**^ (Ncm)				
Mean	13.14	15.82	19.92^a^	25.20^b^
SD	5.67	4.76	6.83	5.01

*ST: SLA Ti; BI-ST: rhBMP-2-immobilized ST; BT-ST: rhBMP-2-treated ST; R-ST: rGO-coated ST

**MRT Maximum Removal Torque

### Histological and histomorphometric findings

**Fig. 6:**
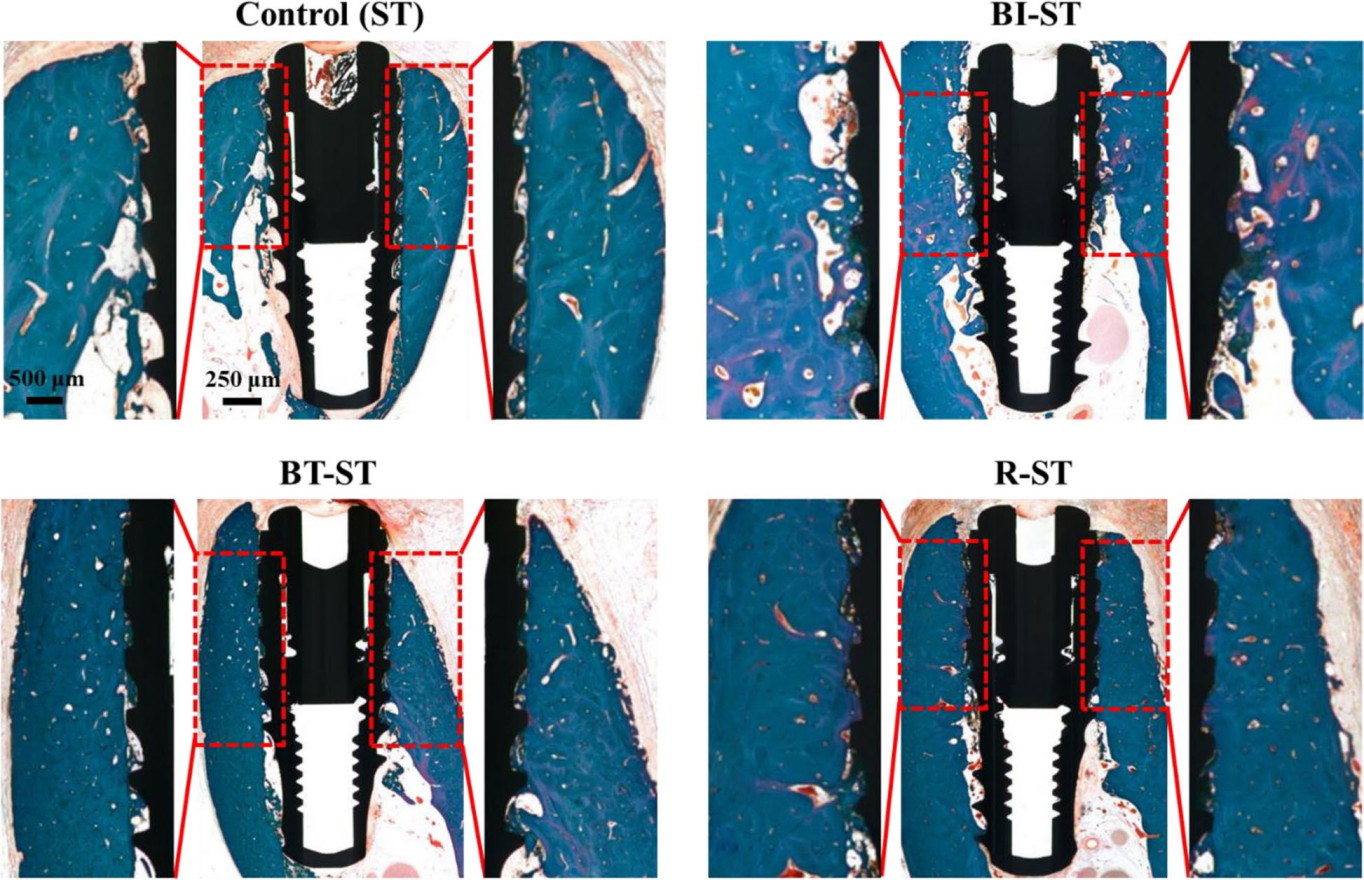
Histological section of peri-implant dehiscence defect areas from all the experimental groups (control, BI-ST, BT-ST, and R-ST) at 8 weeks after surgery. The ROI for measurements of BIC length and ITBD is shown with enlarged view (Goldner Trichrome staining)

The histological measurement was helpful to identify the components involved in bone tissue regeneration during the 8-week healing period. The expanded view of the BIC clearly shows that the BT-ST and R-ST groups have more osseointegrated areas than the BI-ST and control groups comparatively (Fig. 6). A matured bone growth was observed in the R-ST implant-placed animal group, while relatively large voids and osteoid regions transforming into new bone were found in other groups. For a better understanding, the upper three threads of ROI in the control were comprehensively demonstrated in Fig. S6, along with the BIC site, implant surface, and biological molecules associated with bone tissue regeneration. The BIC and ITBD ratios were measured from the length (red line) and area (yellow shade), respectively, of three thread portions of the implant at the defect site [40]. The results of histomorphometric variables with SD were summarized in Table 1. Both BT-ST and R-ST groups showed a significant difference compared to the control and BI-ST groups in terms of BIC (%) and ITBD (%).

## Discussion

The present study aims to evaluate the bone tissue regeneration ability of different Ti surfaces, which was modified by SLA treatment and subsequently fabricated by biocompatible rGO (coated) and rhBMP-2 (immobilized or treated). It was reported that Ti implants with rougher surfaces provide a larger surface area between implant and bone, enhance friction, improve osseointegration, and ultimately augment their early stability [41]. SLA is the representative method that presents astonishing implant fixation and a higher success rate as compared to the early mechanically treated surfaces [42]. However, the lack of strategies for stimulating dental tissue regeneration has been acknowledged as a clinical challenge. In the present work, we fabricated the R-ST (i.e. rGO-coated SLA Ti) implants and compared the effects of different surface treatments of Ti implants on osseointegration to develop a novel strategy for promoting dental tissue regeneration.

We first optimized the rGO concentration in coating process on the R-ST, and the optimized concentration (100 μg/mL) of rGO solution was found to be capable of providing a suitable surface for cell growth through the examination of surface chemistry and in vitro cell behavior analyses (Table S1 and Fig. 3). Though dose-dependent changes have been observed for the physicochemical characterizations within the manufacturing conditions, the optimized R-ST disc showed a promising result to the investigated hMSCs than other concentrated samples. The series of spectral, diffraction, and microscopic analyses were helpful to identify the surface properties of the R-ST discs (Figs. 1 and 2). According to the Raman spectra, the I_D_/I_G_ ratio revealed that rGO was successfully synthesized without the use of toxic chemicals; instead, we utilized a phytochemical (EGCG), one of the most abundant polyphenols found in green tea [43], in the reduction step. The I_2D_/I_G_ ratio of less than 1 indicates a multi-layered graphitic structure with the presence of few defect sites, revealed by a less intensity of the 2D band to G band [25, 28]. Hence, we infer that the rGO synthesized using phytochemical has many layers with few defects in its crystal structure as evidenced by the low I_2D_/I_G_ (0.37 ± 0.04) value (Figs. 1A and S3A). On the other hand, the Ti 2p peak disappeared in XPS of the R-ST while the FTIR spectrum of the R-ST exhibited strong characteristic peaks of rGO, suggesting that the surface of Ti material was well coated by rGO during fabrication (Figs. 1B, C and S3B). From the EBSD measurement, the rGO coating has no critical effect on the regular and ultrafine-grained surface of Ti discs (Fig. 2A). Meanwhile, the presence of rGO on the R-ST surface naturally influenced an increase in the surface roughness (R_*a*_) value by adding the nanofilms (Fig. 2B). It has been reported that the micro/nanoscale surface roughness can enhance and direct the growth of bone-related cells while achieving osseointegration at the osseous site [44]. The reduction in average grain size and smooth surface in the measured area for the R-ST samples revealed that the microstructure of the Ti disc was improved due to effective rGO coating. Because rGO possesses abundant oxygen-containing functional groups on its basal plane, the rGO coating could increase the hydrophilicity of the cell-interfaced surface in the case of the R-ST samples (Fig. 2C). This is an important parametric feature to determine the bioactive capability of the implant surface with the microenvironment to the cells and related many biological macromolecules. Moreover, the provided implant surface plays an important role in ensuring biocompatibility and promoting the early stage of osseointegration by affecting cellular growth and accelerating adhesion of plasma proteins including prothrombin [45]. Previous studies revealed that osteoblasts cultured on hydrophilic surfaces exhibited higher levels of osteogenic differentiation markers such as ALP and OCN when compared to hydrophobic surfaces [46]. Altogether, as the coating of rGO on the Ti surface can effectively improve the surface properties of Ti material, we envisage that the resulting surface bioactivity of the implants would aid for effective in vivo osseointegration during bone tissue regeneration application.

As presented, the osteogenic capacity of hMSCs on the varied Ti discs was demonstrated to compare cell attachment, proliferation, ALP activity, and extracellular mineralization. Within the controlled experimental conditions, the R-ST group obviously showed the highest beneficial effects on the cellular behaviors. So far, there have been extensive studies on developing functionalized Ti surfaces that can enhance osteogenesis and accelerate bone tissue regeneration using various biomolecules. For example, Ti surface immobilized with siRNA targeting the MIR31HG gene (siMIR31HG) was found to promote the osteogenic differentiation of MSCs, and the siMIR31HG-functionalized surface could induce larger ALP production, compared to the siRNA control with a thermal alkali-treated surface [47]. It was reported that laser-treated Ti discs with a nano-scaled topography could reduce the proliferation of human umbilical cord MSCs but increase osteogenic differentiation [48]. More recently, it was discovered that Ti surfaces functionalized with exosomes derived from MSCs could effectively interact with MSCs and rapidly promote MSC adhesion and proliferation [49]. The surfaces of immobilized exosomes and the combination of immobilized and suspended exosomes increased the surface coverage of cells as compared to the Ti control surface. Likewise, bioinspired material 3,4-dihydroxy-L-phenylalanine (DOPA)-coated Ti surface exhibited enhancement of cellular adhesion and spreading compared to the uncoated control [50]. It was impressive that simple DOPA coating significantly enhanced the ALP activity of MSCs compared to control cells at day 7 of the investigation. Nevertheless, some researchers are still skeptical of biomolecules or growth factors due to the possibility of their unpredictable side effects, such as ectopic bone formation and postoperative inflammation [20]. In contrast, our surface modification strategy of the R-ST can be one possible alternative at a high level to promote cell growth and osteogenic differentiation without any biochemical factors, which suggests that it has an unprecedented impact on the development classified as a novel surface modification method for dental applications (Figs. 4 and 5).

Importantly, to validate the in vivo performance of the R-ST implant, we examined the effects of the R-ST implants on osseointegration and new bone formation in a beagle dog model (Tables 1 and 2, and Fig. 6). During the healing period of implant sites, no implant failure was evaluated, ensuring all groups maintained primary stability, which is a prerequisite characteristic of osseointegration. Since osseointegration integrates vital bone with the surface of the implant by a direct structural and functional connection, the implant contact site acts as the platform to interact with bone growing cells during bone tissue regeneration [2]. On this, the volumetric and histomorphometric results clearly demonstrated that the R-ST implant exhibited larger NBV, BIC, and ITBD values than other groups (Table 1). Similar work has been reported on dental tissue regeneration by extracting teeth from the mandible of beagle dogs using a 3D printed scaffold loaded with rhBMP-2 [51], which revealed NBV of the BMP-treated groups (10.08 ± 2.40 mm^3^) was significantly higher than other control groups (6.30 ± 2.90 mm^3^). The percentage of the BIC ratio was also found to be 22.61 ± 6.92% and 51.29 ± 14.64% for the control and BMP group, respectively. Moreover, various volumetric and histomorphometric parameters of collagen membrane (CM) and electron beam irradiated bacterial cellulose membrane (EI-BCM) were evaluated in peri-implant dehiscence defects of beagle dogs, in which NBV and BIC values of CM and EI-BCM treated groups were limited up to 3 mm^3^ and 55%, respectively [52]. In another study, the effects of immobilization of rhBMP-2 on Ti surfaces on osseointegration and bone regeneration were investigated [24]. The maximum ITBD value of recombinant human platelet-derived growth factor-BB and rhBMP-2 immobilized Ti implants was 69.22 ± 3.96%, which was significantly greater than that of pure Ti discs (54.90 ± 7.24%). On the other hand, it was reported that the anodized TiO_2_ nanotube coated with a certain thickness of the PLGA layer was an ideal and suitable rhBMP-2 carrier to enhance osseointegration [53]. In our present work, the R-ST implant showed a significant improvement in dental tissue regeneration (NBV = 41.84 ± 4.27 mm^3^, BIC = 89.45 ± 5.32%, and ITBD = 88.58 ± 5.83%), while even the control group (ST implant) has shown betterment in terms of NBV, BIC, and ITBD. Moreover, these results well agreed with those of the removal torque test, showing that the highest MRT value (25.20 ± 5.01 Ncm) was recorded in the R-ST implant (Table 2).

It has been well-known that osteoblasts secrete an unmineralized organic matrix called osteoid in the initial phase, which turns into mineralized bone tissue during the maturation phase. The combination of the osteoid and mineralized bone matrix represents a total bone tissue and the osteoid formation is facilitated by the increased surface wettability of the implant [40]. The observation of abundant osteoid mass and void in the intact Ti implant indicates that the ST implant-treated animal group did not recover completely at the end of the implantation period. Meanwhile, the R-ST implant-treated animal group has a complete mineralized bone tissue forming a dense network close to the implant surface (Fig. 6). Based on the results of physicochemical characterizations and biological evaluations, we might conclude that the R-ST implant has the desired surface properties of Ti implant, such as surface topography, roughness, and molecular wettability, which influenced largely cell attachment and proliferation, ALP activity, extracellular matrix mineralization, and expression of osteogenesis-related genes and protein. Furthermore, our findings in vivo demonstrated that the R-ST implant might be employed as a novel dental implant material that can provide an amicable microenvironment for the integration of regenerating tissues and the implant site as evidenced by a promising osseointegration profile and the accelerated bone tissue regeneration.

## Conclusions

In the present study, the surface of ST-based discs and implants was modified with different substances such as rhBMP-2 and rGO, and then characterized along with the unmodified control. We found that the R-ST exhibited excellent surface properties that can promote in vitro osteogenic differentiation and accelerate in vivo osseointegration. This current discovery of the potential of the rGO-coated sample is of paramount importance in the context of the development of functional bionanomaterials to guide and promote dental tissue regeneration. Furthermore, it is expected that this study could pave the way to opt for promising implant surface based on those in vitro and in vivo analyses. Our fascinating results can be summarized as follows:

- The rGO-coated Ti surface has excellent biocompatibility and superior ability to absorb the exogenous proteins, which can elicit robust cellular responses.
- The rGO-coated Ti surface significantly promotes cell growth and osteogenic differentiation without any osteogenic factors.
- The rGO-coated implant significantly accelerates the osseointegration and dental tissue regeneration in vivo.

Taken together, these observations, which could have general significance, suggest that the rGO-coated Ti can be a promising candidate for developing of futuristic dental and orthopedic implants to accelerate bone regeneration and osseointegration.

## Supplementary Information

**Additional file 1: Table S1.** Optimization of rGO coating on SLA Ti (ST) surface (10-1000 μg/mL). Surface properties and cellular behaviors of hMSCs on the R-ST discs were investigated. Data are expressed with mean ± SD (*p* < 0.05^a^, *p* < 0.01^b^, and *p* < 0.001^c^, *n* = 6). **Table S2.** qRT-PCR primer sequences for RUNX2, OCN, OPN, Vinculin, and β-actin. **Fig. S1.** Microscopic and contact angle analyses of R-ST surfaces coated with 10 and 1000 μg/mL of rGO. 3D AFM images of R-ST surfaces coated with (A) 10 and (B) 1000 μg/mL of rGO. Water contact angles (θ) of R-ST surfaces coated with (C) 10 and (D) 1000 μg/mL of rGO. **Fig. S2.** (A) FL intensity and (B) immunofluorescence staining of rhBMP-2 immobilized on ST surface (red FL from TRITC). **Fig. S3.** Spectral analysis of ST and R-ST surfaces coated with a range of rGO concentrations (10, 100, and 1000 μg/mL). (A) Raman and (B) FTIR spectra of ST and R-ST discs. **Fig. S4.** Quantification of the surface protein adsorption on the ST, BI-ST, and R-ST discs. Protein concentrations were determined by the bicinchoninic acid (BCA) assay after incubation with (A) Dulbecco’s phosphate-buffered saline containing 10% fetal bovine serum (FBS), (B) MSC basal media (without any supplements or FBS), and (C) complete media (with supplements and 10% FBS) for 24 h at 37°C. (D) Immunofluorescence images (green FL from FITC) of adsorbed proteins on the surface of each disc after incubation with (A). The data are expressed as the mean ± SD (*n* = 6). An asterisk (*) denotes a statistically significant difference compared to the control (ST), *p* < 0.05. **Fig. S5.** (A) Clinical photographs depicting the flattening of alveolar bone, (B) trimming alveolar ridge into a flat ridge and making drilled holes for the implant placement and (C) implants inserted into the alveolar ridge by surgical procedures. The micro-computed tomography (μ-CT) images of mesiodistal section of all the implant sites were reconstructed. (D) The region of interest (ROI, shown in blue shade) with implant contact site displayed with a width of 1.0 mm and height of 4.0 mm. (E) 3D reconstructed μ-CT image of occlusal section of the implant site (left) and ROI around the implant (right). **Fig. S6.** (A) Histological specimen of ST implant (control). (B) A magnified view of the upper three threads of a region of interest (ROI), from which histomorphometric parameters such as intra-thread bone density (ITBD) area and bone-to-implant contact (BIC) length were measured (A symbol, ‘I’ represents an implant fixture.). (C) ITBD area shown with yellow shade. (D) The boundary line (red) has been drawn over the implant surface. The dotted line indicates the place of implant contact with the regenerated bone tissue (BIC) whereas the solid line implies the void left. (E) The biological components involved in the bone tissue regeneration process are indicated as follows: solid arrow (void); dashed arrow (osteoid); new bone (*).

## Data Availability

All data generated or analyzed during this study are included in this published article.

## Abbreviations

AFM: Atomic force microscopy
ALP: Alkaline phosphatase
ANOVA: Analysis of variance
APTES: (3-Aminopropyl)triethoxysilane
ARS: Alizarin red S
BCA: Bicinchoninic acid
BIC: Bone-to-implant contact
BI-ST: rhBMP-2-immobilized ST
BM: Basal media
BMP: Bone morphogenetic proteins
BSA: Bovine serum albumin
BT-ST: rhBMP-2-treated ST
CCK-8: Cell counting kit-8
DAPI: 4′,6-Diamidino-2-phenylindole
DI: De-ionized
DOPA: 3,4-Dihydroxy-L-phenylalanine
DPBS: Dulbecco’s phosphate-buffered saline
EBSD: Electron backscattered diffraction
EDAX: Energy-dispersive X-ray
EGCG: Epigallocatechin-3-*O*-gallate
ITBD: Intra-thread bone density
FBS: Fetal bovine serum
FITC: Fluorescein isothiocyanate
FL: Fluorescence
FTIR: Fourier transform infrared
GO: Graphene oxide
hMSCs: Human mesenchymal stem cells
MRT: Maximum removal torque
μ-CT: Micro-computed tomography
NBV: New bone volume
OCN: Osteocalcin
OPN: Osteopontin
qRT-PCR: Quantitative reverse transcription polymerase chain reaction
rGO: Reduced GO
rhBMP-2: Recombinant human BMP-2
ROI: Region of interest
R-ST: rGO-coated SLA Ti
RT: Room temperature
RUNX2: Runt-related transcription factor 2
SD: Standard deviation
SLA: Sandblasted, large-grit, and acid-etched
ST: SLA Ti
Ti: Titanium
TRITC: Tetramethylrhodamine isothiocyanate
XPS: X-ray photoelectron spectra
