# Supplementary material for: Enhanced osseointegration of dental implants with reduced graphene oxide coating
Source: Biomater Res. 2022 Mar 21;26:11. doi: 10.1186/s40824-022-00257-7 (PMC8935794; doi:10.1186/s40824-022-00257-7)
Supplement: Supplementary file 1 — Additional file 1: Table S1. Optimization of rGO coating on SLA Ti (ST) surface (10-1000 μg/mL). Surface properties and cellular behaviors of hMSCs on the R-ST discs were investigated. Data are expressed with mean ± SD (p < 0.05a, p < 0.01b, and p < 0.001c, n = 6). Table S2. qRT-PCR primer sequences for RUNX2, OCN, OPN, Vinculin, and β-actin. Fig. S1. Microscopic and contact angle analyses of R-ST surfaces coated with 10 and 1000 μg/mL of rGO. 3D AFM images of R-ST surfaces coated with (A) 10 and (B) 1000 μg/mL of rGO. Water contact angles (θ) of R-ST surfaces coated with (C) 10 and (D) 1000 μg/mL of rGO. Fig. S2. (A) FL intensity and (B) immunofluorescence staining of rhBMP-2 immobilized on ST surface (red FL from TRITC). Fig. S3. Spectral analysis of ST and R-ST surfaces coated with a range of rGO concentrations (10, 100, and 1000 μg/mL). (A) Raman and (B) FTIR spectra of ST and R-ST discs. Fig. S4. Quantification of the surface protein adsorption on the ST, BI-ST, and R-ST discs. Protein concentrations were determined by the bicinchoninic acid (BCA) assay after incubation with (A) Dulbecco’s phosphate-buffered saline containing 10% fetal bovine serum (FBS), (B) MSC basal media (without any supplements or FBS), and (C) complete media (with supplements and 10% FBS) for 24 h at 37°C. (D) Immunofluorescence images (green FL from FITC) of adsorbed proteins on the surface of each disc after incubation with (A). The data are expressed as the mean ± SD (n = 6). An asterisk (*) denotes a statistically significant difference compared to the control (ST), p < 0.05. Fig. S5. (A) Clinical photographs depicting the flattening of alveolar bone, (B) trimming alveolar ridge into a flat ridge and making drilled holes for the implant placement and (C) implants inserted into the alveolar ridge by surgical procedures. The micro-computed tomography (μ-CT) images of mesiodistal section of all the implant sites were reconstructed. (D) The region of interest (ROI, shown in blue shade) wi [file 40824_2022_257_MOESM1_ESM.docx]

***Supplementary Material***

**Enhanced osseointegration of dental implants with reduced graphene oxide coating**

Yong Cheol Shin^1,2†^, Ji-Hyeon Bae^3†^, Jong Ho Lee^4†^, Iruthayapandi Selestin Raja^5^, Moon Sung Kang^1^, Bongju Kim^6^, Suck Won Hong^1^*, Jung-Bo Huh^3^*, Dong-Wook Han^1,5^*

*^1^Department of Cogno-Mechatronics Engineering, College of Nanoscience & Nanotechnology, Pusan National University, Busan 46241, South Korea*

*^2^Department of Biomedical Engineering, The University of Texas at Austin, Austin, TX 78712, USA*

*^3^**Department of Prosthodontics,* *Dental Research Institute,* *Dental and Life Science Institute, Education and Research Team for Life Science on Dentistry, School of Dentistry, Pusan National University,* *Yangsan 50612, South Korea*

*^4^Daan Korea Co., Ltd., Busan 47149, South Korea*

*^5^BIO-IT Foundry Technology Institute, Pusan National University, Busan 46241, South Korea*

*^6^Dental Life Science Research Institute / Innovation Research & Support Center for Dental Science, Seoul National University Dental Hospital, Seoul 03080, South Korea*

***** Corresponding authors.

*Email addresses*: swhong@pusan.ac.kr (S.W. Hong), huhjb@pusan.ac.kr (J.-B. Huh), nanohan@pusan.ac.kr (D.-W. Han)

^†^ These authors contributed equally to this work.

**Table S1.** Optimization of rGO coating on SLA Ti (ST) surface (10-1000 μg/mL). Surface properties and cellular behaviors of hMSCs on the R-ST discs were investigated. Data are expressed with mean ± SD (*p* < 0.05^a^, *p* < 0.01^b^, and *p* < 0.001^c^, *n* = 6).

| **Variables** | **Control (ST)** | **R-ST (μg/mL, rGO concentration)** | | |
| --- | --- | --- | --- | --- |
|  |  | **10** | **100** | **1000** |
| **Arithmetic average**  **roughness (R*_a_*, nm)** | 79.3 ± 0.9 | 76.4 ± 1.2 | 87.1 ± 1.8 ^b^ | 104.4 ± 2.3 ^c^ |
| **Contact angle (°)** | 70.5 ± 1.9 | 58.2 ± 6.0 ^a^ | 53.6 ± 11.9 ^b^ | 48.5 ± 3.7 ^b^ |
| **Surface energy (mN/m)** | 42.0 ± 1.4 | 50.2 ± 4.8 ^b^ | 53.4 ± 8.4 ^c^ | 55.4 ± 3.9 ^c^ |
| **Cell attachment (%)** | 100 ± 1 | 123 ± 5 ^b^ | 146 ± 3 ^c^ | 119 ± 8 ^b^ |
| **Cell proliferation (%) at 21 d** | 406 ± 52 | 417 ± 29 | 482 ± 17 ^b^ | 214 ± 41 ^c^ |
| **ALP activity**  **(nmol/mL/min) at 14 d** | 14 ± 5 | 67 ± 7 ^c^ | 95 ± 7 ^c^ | 22 ± 6 |
| **Mineralized nodule (%) at 21 d** | 298 ± 32 | 3282 ± 26 ^c^ | 4612 ± 21 ^c^ | 1010 ± 24 ^c^ |

**Table S2.** qRT-PCR primer sequences for RUNX2, OCN, OPN, Vinculin, and β-actin.

| **Gene** | Forward sequence (5′ → 3′) | Reverse sequence (5′ → 3′) |
| --- | --- | --- |
| **RUNX2** | CACTACCCAGCCACCTTTAC | GGATGCTGACGAAGTACCATAG |
| **OCN** | CCAAGCAGGAGGGCAATAA | TCGTCACAAGCAGGGTTAAG |
| **OPN** | ACGACGATGATGACGATGATG | GTAGGGACGATTGGAGTGAAAG |
| **Vinculin** | GGCAGAGGTAGTGGAAACTATG | CTCCTGCTGTCTCTCATCAATC |
| **β-actin** | CGTTCAATACCCCAGCCATG | GACCCCGTCACCAGAGTCC |


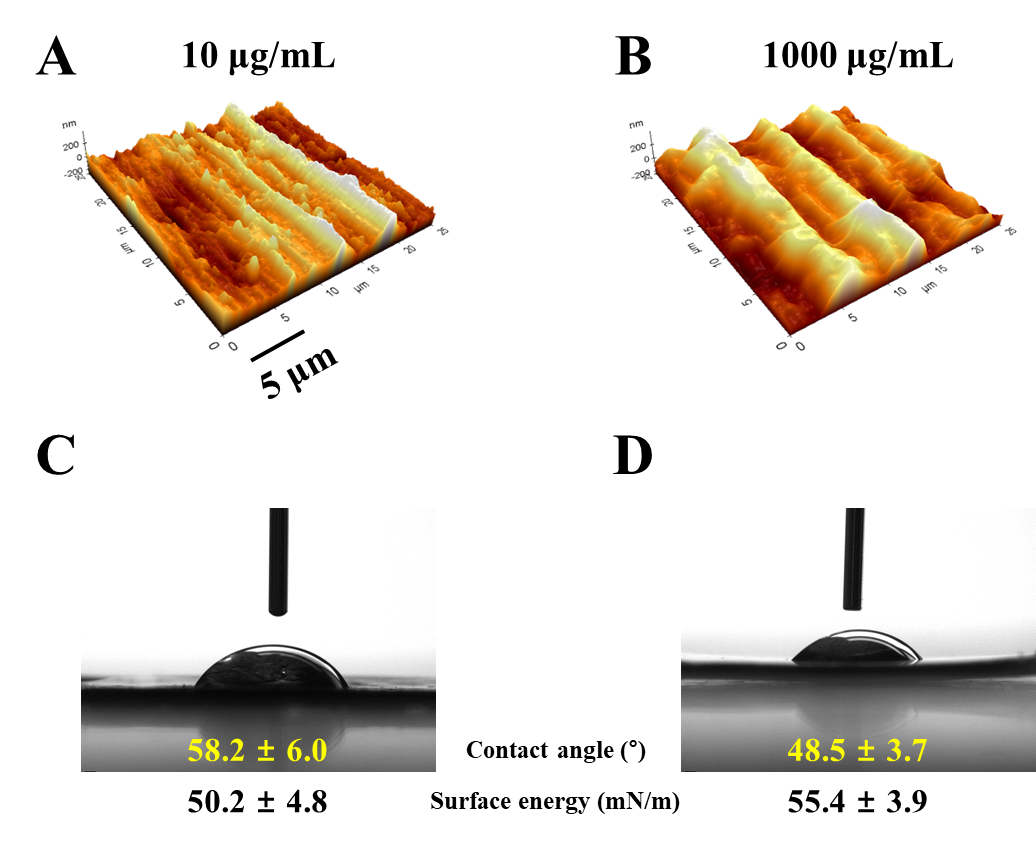


**Fig. S1.** Microscopic and contact angle analyses of R-ST surfaces coated with 10 and 1000 μg/mL of rGO. 3D AFM images of R-ST surfaces coated with (A) 10 and (B) 1000 μg/mL of rGO. Water contact angles (θ) of R-ST surfaces coated with (C) 10 and (D) 1000 μg/mL of rGO.


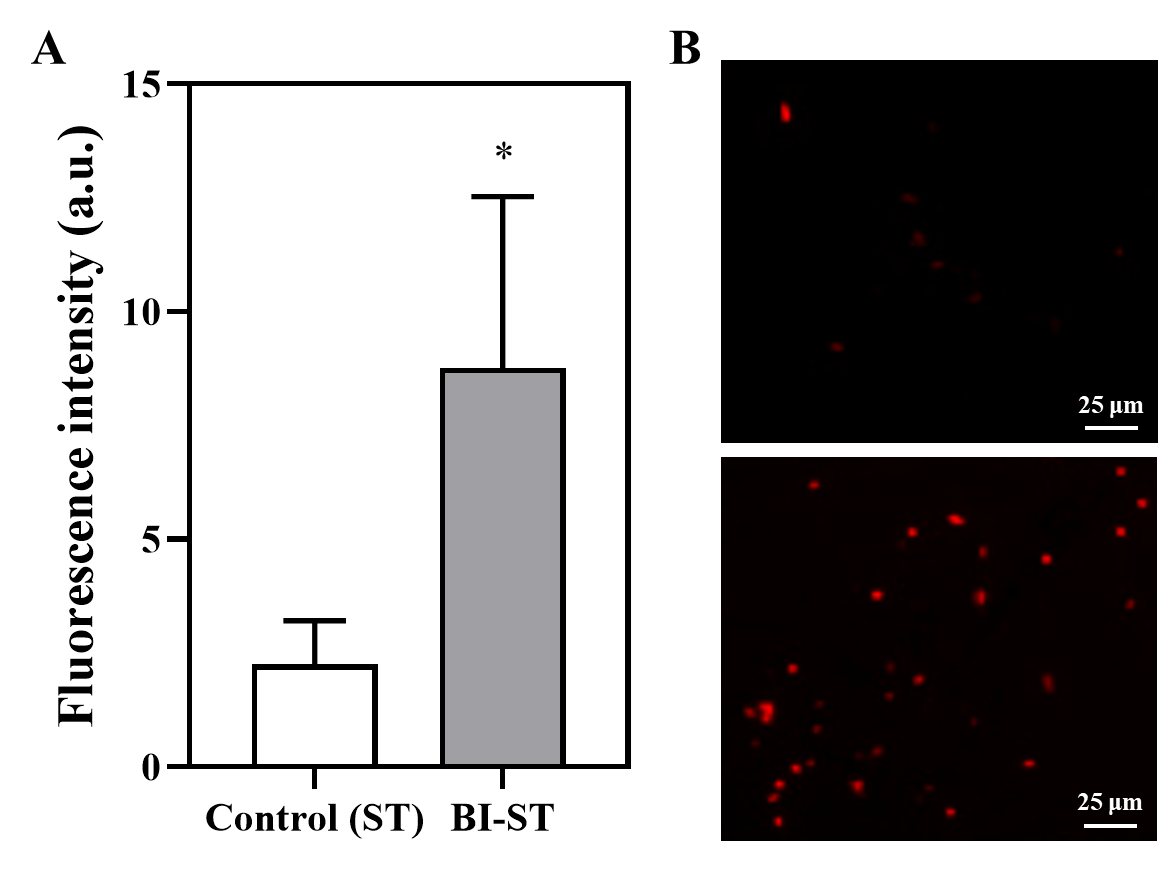


**Fig. S2.** (A) FL intensity and (B) immunofluorescence staining of rhBMP-2 immobilized on ST surface (red FL from TRITC).


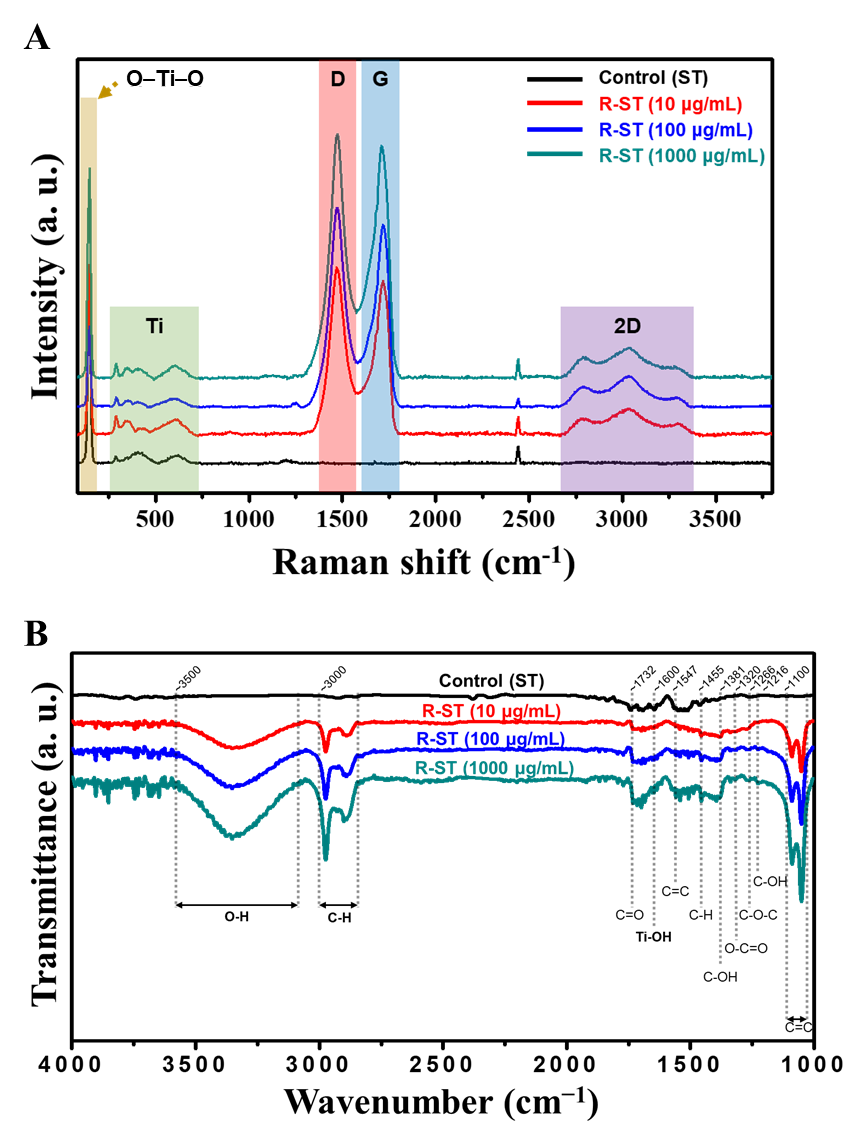


**Fig. S3.** Spectral analysis of ST and R-ST surfaces coated with a range of rGO concentrations (10, 100, and 1000 μg/mL). (A) Raman and (B) FTIR spectra of ST and R-ST discs.


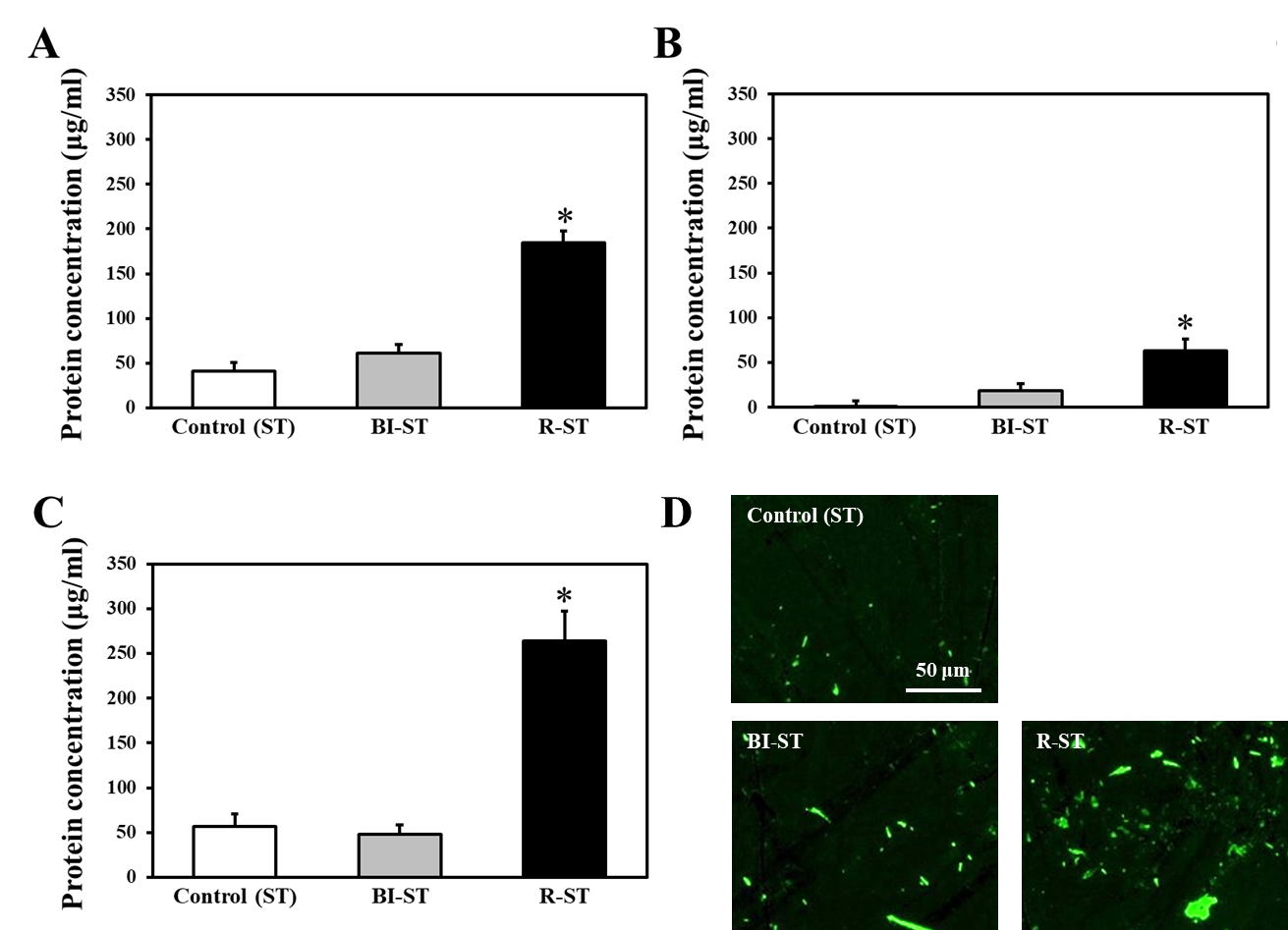


**Fig. S4.** Quantification of the surface protein adsorption on the ST, BI-ST, and R-ST discs. Protein concentrations were determined by the bicinchoninic acid (BCA) assay after incubation with (A) Dulbecco’s phosphate-buffered saline containing 10% fetal bovine serum (FBS), (B) MSC basal media (without any supplements or FBS), and (C) complete media (with supplements and 10% FBS) for 24 h at 37°C. (D) Immunofluorescence images (green FL from FITC) of adsorbed proteins on the surface of each disc after incubation with (A). The data are expressed as the mean ± SD (*n* = 6). An asterisk (*) denotes a statistically significant difference compared to the control (ST), *p* < 0.05.


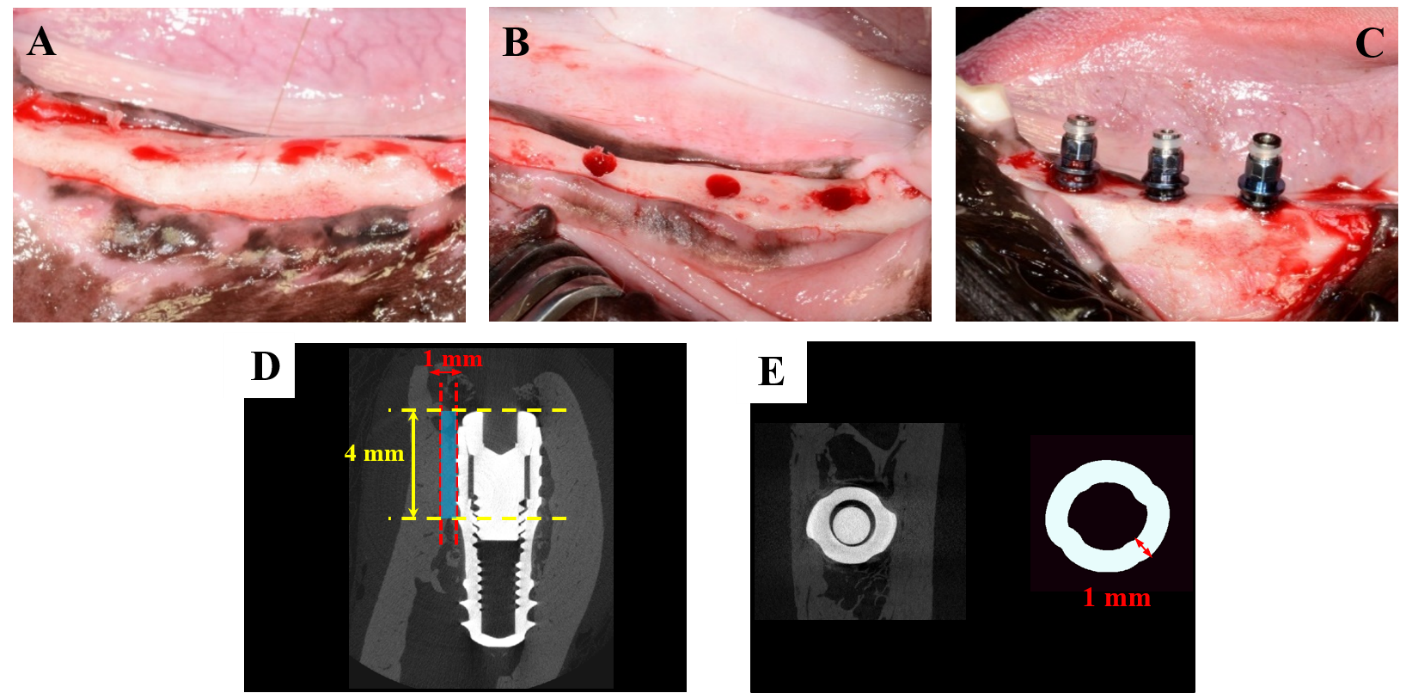


**Fig. S5.** (A) Clinical photographs depicting the flattening of alveolar bone, (B) trimming alveolar ridge into a flat ridge and making drilled holes for the implant placement and (C) implants inserted into the alveolar ridge by surgical procedures. The micro-computed tomography (μ-CT) images of mesiodistal section of all the implant sites were reconstructed. (D) The region of interest (ROI, shown in blue shade) with implant contact site displayed with a width of 1.0 mm and height of 4.0 mm. (E) 3D reconstructed μ-CT image of occlusal section of the implant site (left) and ROI around the implant (right).


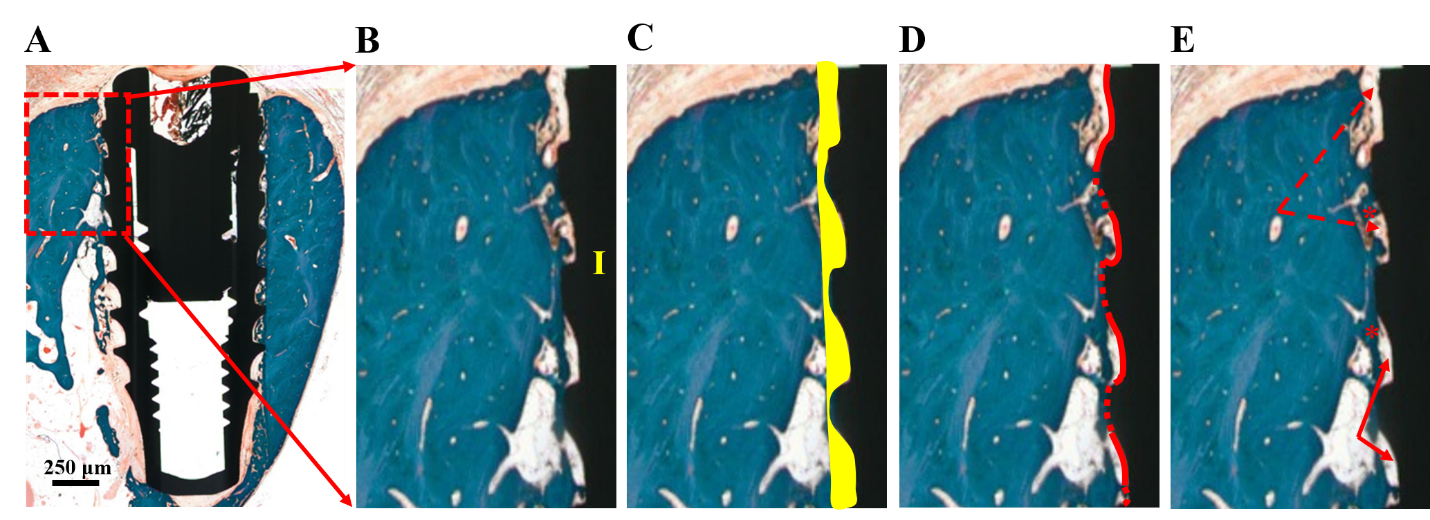


**Fig. S6.** (A) Histological specimen of ST implant (control). (B) A magnified view of the upper three threads of a region of interest (ROI), from which histomorphometric parameters such as intra-thread bone density (ITBD) area and bone-to-implant contact (BIC) length were measured (A symbol, ‘I’ represents an implant fixture.). (C) ITBD area shown with yellow shade. (D) The boundary line (red) has been drawn over the implant surface. The dotted line indicates the place of implant contact with the regenerated bone tissue (BIC) whereas the solid line implies the void left. (E) The biological components involved in the bone tissue regeneration process are indicated as follows: solid arrow (void); dashed arrow (osteoid); new bone (*).
